# Enrichment of medium-quality colostrum by adding colostrum replacer, combined or not with transition milk in the feeding of dairy calves

**DOI:** 10.1038/s41598-024-55757-4

**Published:** 2024-03-06

**Authors:** Ana Paula Silva, Amanda M. Cezar, Ariany F. de Toledo, Marina G. Coelho, Cristiane R. Tomaluski, Gercino F. Virgínio Júnior, Carla M. M. Bittar

**Affiliations:** 1 Department of Animal Science, “Luiz de Queiroz” College of Agriculture, University of São Paulo, Av. Pádua Dias, N 11, Piracicaba, São Paulo 1341-900 Brazil; 2 Minas Gerais Agricultural Research Agency, Experimental Field of Montes Claros, Montes Claros, Minas Gerais 39404-128 Brazil

**Keywords:** Early nutrition, Immunoglobulin, Neonate, Nutrition, Animal physiology

## Abstract

Fifty Holstein calves were allocated in randomized blocks and distributed in a 2 × 2 factorial arrangement; (A) two sources of Ig: (1) Control: bovine colostrum (25% Brix); (2) Enriched colostrum: mid-quality bovine colostrum (20% Brix) enriched with colostrum replacer to 25% Brix; and (B) two transition feeding diets: (1) Whole milk (WM): supply of 4 L/day of whole milk for 3 days after the colostrum feeding; and (2) Formulated transition milk (FTM): supply 4 L/day of whole milk enriched with 70 g/L of colostrum replacer for 3 days after the colostrum feeding. Blood samples were collected at 0, 24, 48, and 72 h of age to determine total serum protein (TSP), glucose, non-esterified fatty acids (NEFA), erythrocyte and leukocyte concentrations. IgG was measured at 48 h. During the preweaning period, calves received 6 L/day of whole milk. Blood samples were collected weekly to determine TSP, glucose, and lactate. The colostrum protocols were equally efficient for transfer of passive immunity with IgG concentration at 48 h ≥ 49.6 g/L. Colostrum or transition feeding program did not influence the erythrocyte and leukocyte concentrations. The TSP concentration measured until 72 h was higher for calves fed maternal colostrum. Calves fed milk in the transition period had higher glucose concentrations. Calves receiving bovine colostrum and FTM had higher glucose concentrations in the preweaning period, while the enriched colostrum decreased plasma lactate concentrations. In summary, enrichment of mid-quality colostrum is an alternative in situations of a shortage of high-quality colostrum; however, feeding 4 L/day of FTM only for 3 days after colostrum feeding does not show additional benefits.

## Introduction

Early nutrition throughout the neonatal period significantly influences calf-rearing success and affects long-term health and performance into productive life^1,2^. Colostrum feeding is widely recognized as essential for survival and disease prevention in newborn calves by transferring maternal antibodies, mainly immunoglobulin G (IgG)^3^.

Bovine colostrum is the first secretion produced by the mammary gland after calving, and it is composed of a complex mixture of proteins, lipids, lactose, vitamins, and minerals, which provides the first nutritional components for newborn calves^4^. Besides the nutritional effect, colostrum has a fundamental biological function for neonates due to high concentrations of immunoglobulins, which are necessary to confer passive immunity to the newborn calf with an immature immune system^5^. Indeed, maximizing passive transfer of immunity can increase growth performance during the first month of life^6^, and because of its effect in decreasing not only mortality but also morbidity, feeding high-quality colostrum is recommended^7^. Other bioactive compounds are also present in colostrum, such as insulin-like growth factors (IGF-I and IGF-II), insulin, lactoferrin, lysozyme, and lactoperoxidase^8^. Although the current research recommends providing colostrum at a minimum of 22% Brix^7^, corresponding to 50 mg/mL of immunoglobulins, the total IgG intake recommendations have stimulated dairy farmers to provide colostrum with higher Brix^9^ and or a second meal to dairy calves at birth^10^. It is worth to noting that colostrum replacers (CR) differ from colostrum supplements, since CR contains high mass of IgG, usually > 100 g/dose, and all the other nutrients and bioactive components^11^. However, whether the bioactive components remain functional after the spray-dry process used to make colostrum powder from fresh colostrum is not known.

In addition to colostrum feeding, providing transition milk is an early feeding management important in calf rearing, and is recommended by National Academies of Science, Engineering, and Medicine (NASEM)^12^. Transition milk is defined as milk collected after colostrum milking, between the second and sixth milking after the calf birth^5,13^, and still contains considerable concentrations of bioactive compounds^14–16^. Thus, such feeding management, in addition to increasing the nutrient supply due to the higher fat and protein contents in transition milk, extends the intake of IgG and bioactive compounds, positively affecting intestinal development and, consequently, the health of neonates^15,17,18^. However, dairy farms don’t always have the resources to feed transition milk to newborn calves, due to either the logistics in the milking parlor or the storage problems associated for latter feeding.

Although the importance of early nutrition is unquestionable^3,7,8,10^, there are situations when dairy farms might not have sufficient quality or quantity of colostrum, even though storing surplus colostrum ≤ 4 °C for a few days is an option, but freezing colostrum is always recommended^19^. Colostrum replacer is an alternative to be exclusively fed to the newborn when bovine maternal colostrum is unavailable or if the quality is compromised by low IgG levels or the presence of pathogens^20–23^. However, the required IgG mass may be achieved by feeding a higher volume when IgG is low in the available colostrum. In addition, colostrum replacer can supplement the liquid diet in the first days of life to mimic maternal transition milk^24^. Providing maternal transition milk or milk replacer supplemented with colostrum replacer promoted higher average daily gain (ADG) compared to providing milk replacer from day 2 to day 4^24^, and compared to providing whole milk from day 2 to 14 of life^25^. Thus, colostrum replacer could be added to medium-quality colostrum (18 to 21% Brix), and also added to the liquid diet in the first days of life to mimic maternal transition milk. This combination in the initial feeding of dairy calves could be an alternative on dairy farms.

Considering this information, we hypothesized that the enrichment of mid-quality colostrum by adding colostrum replacer would increase the amount of IgG and bioactive compounds, and provide transfer of passive immunity to dairy calves similar to high-quality maternal colostrum; moreover, our second hypothesis is that associating either colostrum feeding (high-quality or enriched colostrum) to the feeding of mixture of colostrum replacer in whole milk (formulated transition milk) improves the health and performance of dairy calves. Therefore, the following study aimed to evaluate whether correcting medium-quality colostrum by using colostrum replacer, combined or not with feeding a formulated transition milk, affects the transfer of passive immunity, health, and performance of dairy calves.

## Material and methods

The study was conducted from January to May 2022 at calf facilities in the Department of Animal Science of the "Luiz de Queiroz" College of Agriculture—ESALQ/USP, in Piracicaba-SP. All the experimental procedures were approved following the ethics by the Institutional Animal Care and Use Committee in the "Luiz de Queiroz" College of Agriculture, University of São Paulo, Brazil (Protocol no. 6445260221). We confirm that this study was carried out in compliance with the Animal Research: Reporting of In Vivo Experiments (ARRIVE) guidelines and all methods were performed in accordance with the relevant guidelines and regulations.

### Facilities and animals

This study used fifty Holstein calves (males n = 37 and females = 13) from the university's dairy herd (only females) and a nearby commercial herd. At birth, the calves were immediately separated from their dams to avoid suckling, as colostrum feeding was controlled and differs among calves. Calves born on commercial herds were transported to the university's calf facilities immediately after birth (transport time ≈ 20 min). All the calves received the colostrum feeding protocol as soon as they arrived at the calf yard. Calves were weighed using a mechanical scale (ICS-300, Coimma Ltda., Dracena, SP, Brazil), and the navels were cleaned and disinfected with 7% iodine. Calves were housed in indoor individual calf housing until 14 days, and subsequently in outside individual wood hutches until 56 days of age. Fresh water and calf starter (24.6% CP, 5.22% EE, 9.6% Ashes, 13.89% NDF, 5.52% ADF, 46.57% NFC; Agroceres Multimix, Rio Claro, SP, Brazil) were provided individually from the first day of life.

### Experimental groups

**Figure 1:**
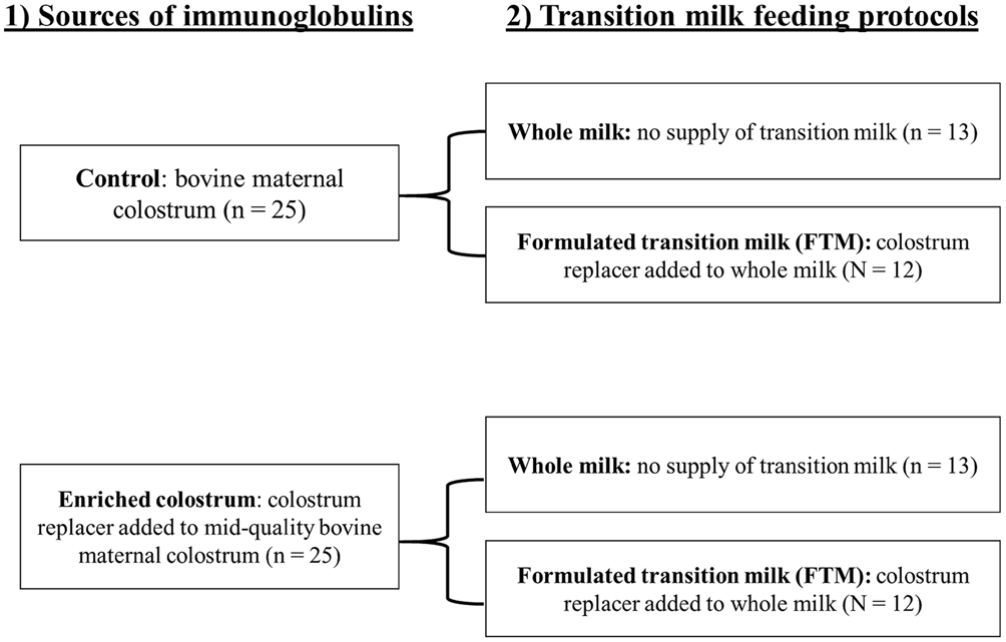
Diagram of the treatments.

The randomized block design was used, and the animals were allocated into blocks according to birth date, birth weight, and sex (which also controls for farm, since the females were from the university herd) and distributed in a 2 × 2 factorial arrangement (two sources of immunoglobulins and two transition milk diets; Fig. 1). The calves were randomly assigned to receive two sources of immunoglobulins: (1) Maternal colostrum: bovine maternal colostrum at minimum 25% Brix, in a volume corresponding to 12% of birth weight; (2) Enriched colostrum: colostrum replacer (CR, SCCL^®^, Saskatoon, Canada) added to mid-quality bovine colostrum (20% Brix) to reach 25% Brix, in a volume corresponding to 12% of birth weight; and two transition milk feeding protocols: (1) Whole milk (WM): no supply of transition milk, this way the calves received 4 L/day of whole milk for 3 days, divided into two meals after colostrum feed; and (2) Formulated transition milk (FTM): supply 4 L/day of whole milk added to 280 g/day (or 60 g IgG/day) of colostrum replacer (70 g/L or 15 g of IgG/L), for 3 days, divided into two meals a day.

The sources of immunoglobulins (Bovine maternal colostrum or Enriched colostrum) were fed within 3 h of birth by bottle or esophageal feeder (4 calves) when the calf had no suckling reflex or did not consume all the amount voluntarily. All calves received colostrum, either high or medium-quality, from a previous formed colostrum bank, thawed in a water bath (55 °C) just before feeding. The colostrum bank was formed during 2 months preceding this study, mid- and high-quality colostrum from cows from the university's dairy herd were frozen and stored in a − 20 °C freezer to be used in this study. For the enriched colostrum treatment, after thawing, the colostrum was standardized with whole milk to obtain a value of 20% Brix and then added CR to achieve 25% Brix. This step was performed quickly and with the aid of an optical Brix refractometer (Lorben, Tonina Comércio Importação e Exportação LTDA, São Paulo, Brazil). Bovine maternal colostrum and enriched colostrum samples were collected in sterilized plastic tubes with 20 mL volume and stored in a freezer (− 20 °C) for later standard plate count (SPC) analysis.

Transition milk or whole milk were fed in the next meal of the calf after colostrum feeding and thereafter until the completion of 6 meals. Thus, calves born in the morning were fed the first transition milk or whole milk meal in the afternoon; and calves born in the afternoon or at night were fed the first meal in the next morning.

### Blood parameters from 0 to 72 h of age

At birth, before colostrum feeding (0 h), and during the transition period from colostrum to liquid diet (24 h, 48 h, and 72 h), blood samples were collected by jugular vein puncture using three different vacuum tubes: (1) containing sodium fluoride as antiglycolytic and potassium EDTA as an anticoagulant to obtain plasma for measurement of glucose, lactate, and non-esterified fatty acids (NEFA); (2) containing potassium EDTA, to evaluate hematocrit, erythrocyte and leukocyte concentrations, as described ahead; and (3) containing clot activator, to obtain serum for measurement of total protein (VACUETTE do Brasil, Campinas, SP, Brazil). The hematocrit was determined in a capillary using a micro-hematocrit centrifuge Model SPIN 1000 (MICROSPIN). Tubes 1 and 3 were centrifuged 2000×*g* for 20 min at 4 °C to obtain plasma and serum, respectively. The determinations of blood parameters were performed in an Automatic System for Biochemistry (Model SBA-200, CELM, Barueri, SP, Brazil). Specific commercial kits were used for the determination of glucose (Ref. 85) and total protein (Ref. 92-250; LABTEST Diagnóstica S.A., Lagoa Santa, Brazil) and for the determination of NEFA (Ref. FA115; RANDOX Laboratories, Life Sciences Ltda, Crumlin, UK).

A serum sample, collected 48 h after colostrum feeding, was used to evaluate the transfer of passive immunity using the Brix refractometer and also by IgG concentration using a commercial ELISA kit (Bovine IgG ELISA Kit, catalog no. E11-118; Bethyl Laboratories Inc., Montgomery, TX, USA). The test was performed according to the manufacturer's instructions. Apparent efficiency of absorption (AEA) was calculated according to the methodology of Quigley and Drewry^26^, using the following equation:

$${\text{AEA IgG }} = \, \left\{ {\left[ {{\text{serum IgG g}}/{\text{L at 48 h }} - {\text{ serum IgG g}}/{\text{L at birth}}} \right] \, \times {\text{ birth weight kg }} \times \, 0.0{9}} \right\}/{\text{ingestion of IgG in grams}}$$

where: 0.09 = plasma volume, calculated as 9% of BW at birth.

Another aliquot of blood of each time point (0 to 72 h) was also used to determine the erythrocyte and leukocyte concentrations. The total erythrocyte count was performed by diluting 20 μL of the blood sample in 4 mL in Gower's Liquid. An average of 20 μL of this diluted sample was pipetted into the side opening of the mirrored Neubauer chamber, causing the liquid to penetrate by capillarity between the chamber and the coverslip. In a microscope, counts were made in five smaller quadrants located in the center of the marked area of the Neubauer chamber, and the number of red blood cells (RBC/μL) was determined by the following equation:

$${\text{RBC}}\, = \,\Sigma {\text{q}}0.{5}.{1}0.{ 2}00.$$

Where: $$\Sigma {\text{q }} = {\text{ sum of the RBC count from 5 quadrants of the Neubauer chamber}}.$$

The total WBC count was performed by diluting 20 μL of the blood sample by 0.4 mL in Turk's Liquid, and a small amount of this diluted sample was pipetted into the side opening of the mirrored Neubauer chamber, causing the liquid to penetrate by capillarity between the chamber and the coverslip. In a microscope, counts were made in the four quadrants located at the ends of the marked area of the Neubauer chamber, and the quantity of WBC/μL was determined by the following equation:

$${\text{WBC/}}\upmu {\text{L}} = \, \left( {\Sigma {\text{q/4}}} \right){.1}0.{2}0$$

where: Σq = sum of the WBC count of the 4 quadrants of the end of the Neubauer chamber.

To determine the differential leukocyte count, blood smears were prepared and stained with Wright's stain (INSTANT PROV, code PA205; NewProv, Pinhais, PR). Differential counting was performed in the Veterinary Clinical Laboratory of the Faculty of Animal Science and Food Engineering, University of Sao Paulo, using a microscope with an immersion objective lens, counting 100 leukocytes and differentiating them by cell morphology, with the aid of a Differential Cell Counter (Model CP 2100, PHOENIX), traversing the lateral edges of the slides in a "zig-zag" path.

### Performance and health

After the initial feeding period (colostrum and FTM or WM during 3 days), calves were fed 6 L/day of whole milk, divided into two meals (7:00 a.m. and 5:00 p.m.) using individual teat buckets, until weaning, any refusal of intake was recorded. Samples of WM were collected weekly for analysis of composition by Fourier transform infrared spectroscopy^27^ and SCC by flow cytometry (Clínica do Leite, Piracicaba, Brazil). The composition of WM was 12.64% total solids, 3.93% fat, 3.29% protein, 4.09% lactose, and SCC of 1272 × 100 cells/mL. The total solids of the WM were used to calculate solids intake for the transition diet and for the TDMI during preweaning. The intake of total solids of FTM was calculated considering 70 g/L of colostrum replacer associated with 12.64% of solids of whole milk, in which the colostrum replacer was diluted, totaling 196.4 g/L of total solids.

The calves had ad libitum access to water and calf starter. Starter was fed daily in the morning, and the leftovers from the previous day were weighed to calculate the daily consumption of concentrate. The preweaning period lasted 8 weeks, and then the experimental period ended. The animals were weighed at birth and weekly until the eighth week of life, always before feeding the morning liquid diet. The average daily gain (ADG) was calculated as the difference of weight between weeks divided by 7 days. Feed efficiency (FE) was calculated as the ratio of ADG and kg of total DMI. The wither height and hip width were measured biweekly, using a ruler (cm), and heart girth was measured using a flexible tape (cm).

The health of the calves was monitored daily following the Calf Heath Scoring Criteria previously published by The University of Wisconsin (Madison) by McGuirk^28^, and all occurrences of diseases and treatments were recorded on individual sheets. The rectal temperature was measured daily using a digital thermometer, and pyrexia was considered when it was above 39.4 °C. The occurrence of diarrhea was monitored daily through the fecal score^29^. Calves were considered to have diarrhea with a fecal score ≥ 2, and two liters of oral rehydration solution (25 g of dextrose, 10 g of sodium bicarbonate, and 5 g of sodium chloride per liter of warm water) were provided 4 h after the morning feeding. Antibiotics were administered as recommended by the veterinarian, only in cases of the calf presenting, besides diarrhea, symptoms such as pyrexia, and/or lethargy. The number of antibiotic treatments were properly recorded, as well as for diarrhea, respiratory disease (RD), and cattle tick fever (CTF, *babesiosis* or *anaplasmosis*).

### Blood parameters at the preweaning period

During preweaning period, blood samples were collected weekly, always two hours after being fed the liquid diet in the morning, to trace the biochemical and metabolites profiles, following the same methodology performed in the first 72 h of life.

### Statistical analysis

The calculation of the sample number was performed using PROC POWER with 90% test power and a significance level of *P* ≤ 0.05, indicating that at least 11 calves per treatment should be enrolled. However, 12 calves were enrolled per treatment when fed FTM and 13 when fed WM. The study was design was a 2 × 2 factorial randomized block, and calves were blocked considering birth date, birth weight, and sex (which also controls for farm, since all the females were from the university herd).

Performance measures (feed intake, ADG, FE, heart girth, withers height, and hip width), erythrocyte count, leukocyte count, and blood parameters (glucose, total protein, lactate, and NEFA) were analyzed as repeated measures over time (hour or week) using the MIXED procedure of the statistical package SAS (version 9.4, SAS Institute Inc, Cary, NC), according to the model:

$$\begin{aligned} {\text{Y}}_{{{\text{ijkl}}}} & = \, \mu \, + {\text{ b}}_{{\text{i}}} + {\text{ F1}}_{{\text{j}}} + {\text{ F2}}_{{\text{k}}} + \, \left( {{\text{F1F2}}} \right)_{{{\text{jk}}}} + {\text{ W}}_{{\text{i}}} + \, \left( {{\text{F1W}}} \right)_{{{\text{jl}}}} \hfill \\ & \quad + \, \left( {{\text{F2W}}} \right)_{{{\text{kl}}}} + \, \left( {{\text{F1F2W}}} \right)_{{{\text{jkl}}}} + {\text{ e}}_{{{\text{ikjl}}}} . \hfill \\ \end{aligned}$$

where, Y_ijk_ = response variable; μ = overall mean; b_i_ = block effect; F1_j_ = effect of factor 1 (Ig source: bovine colostrum vs. enriched colostrum); F2_k_ = effect factor 2 (FTM supply or not); F1F2_jk_ = effect of the F1 × F2 interaction; W_i_ = effect of the age of the animals (hour or days old); F1W_jl_ = effect of the F1 × age interaction; F2W_kl_ = effect of the F2 × age interaction; F1F2W_jkl_ = effect of the F1 × F2 × age interaction; e_ikjl_ = residual error.

Blood metabolite concentrations at birth were used as a covariate for the analysis of each metabolite during the first 72 h of life. Hematocrit was tested as a covariate for the metabolite concentrations evaluated weekly but it was not significant and thus was not included in the model.

The covariance matrices "compound symmetry, heterogeneous compound symmetry, autoregressive, heterogeneous autoregressive, unstructured, banded, variance components, toeplitz, antidependence, and heterogeneous Toeplitz" were tested and defined according to the smallest value obtained for Akaike's Information Criterion (AICC).

Transfer of passive immunity variables (serum Brix, serum IgG, and AEA) and health variables (days with pyrexia, days with diarrhea, and the number of treatments for diarrhea, respiratory illness, and CTF) were analyzed as unrepeated variables using the following statistical model:

$${\text{Y}}_{{{\text{ijk}}}} = \, \mu \, + {\text{ b}}_{{\text{i}}} + {\text{ F1}}_{{\text{j}}} + {\text{ F2}}_{{\text{k}}} + \, \left( {{\text{F1F2}}} \right)_{{{\text{jk}}}} + {\text{ e}}_{{{\text{ikj}}}} .$$

where, Y_ijk_ = response variable; μ = overall mean; b_i_ = block effect; F1_j_ = effect of factor 1 (Ig source: bovine colostrum vs. enriched colostrum); F2_k_ = effect factor 2 (FTM supply or not); F1F2_jk_ = effect of F1 × F2 interaction. The least squares method (LSMEANS) was used to compare means with a significance level of 5%. A tendency was declared at 0.05 ≤ *P* ≤ 0.10.

## Results

### Transfer of passive immunity and FTM intake

Immunoglobulin intake, IgG concentration, Brix (Brix > 9.4%), and AEA of IgG were not affected by colostrum sources (Table 1). The total volume of the transition diet consumed after colostrum feeding tended to be higher for calves fed WM (*P* < 0.06); however, total solids intake was higher for calves fed FTM group (*P* < 0.001), regardless of colostrum feeding protocol.

**Table 1:** Transfer of passive immunity and total solids intake in the first 72 h of life of calves receiving different colostrum and transition milk feeding protocols.

Item	Treatments				SEM^2^	P-value^3^		
	Bovine colostrum		Enriched colostrum					
	FTM^1^	WM	FTM	WM		C	T	C × T
Ig intake, g	518.87	537.45	506.49	507.53	11.477	0.1349	0.4913	0.5205
Serum Brix, % at 48h	10.07	10.49	9.75	10.42	0.299	0.5217	0.1886	0.6696
Serum IgG, g/L at 48h	50.99	51.14	49.60	51.49	3.425	0.8561	0.7289	0.7565
AEA^4^, %	30.45	29.80	30.20	31.30	1.982	0.7497	0.9088	0.6519
Transition diet intake								
Total volume, L	10.79	11.30	11.07	11.25	0.240	0.2973	0.0584	0.8826
Total solids, Kg	2.12^a^	1.43^b^	2.18^a^	1.45^b^	0.037	0.2917	0.0001	0.6635

^1^Formulated transition milk, Whole milk; ^2^Standard error of the mean; ^3^C = effect of colostrum feeding protocol; T = effect of transition feeding protocol; C × T = effect of interaction between colostrum and transition feeding protocol; ^4^Apparent efficiency of absorption; ^a,b^Lower case letters denote effect of transition protocol;

### Erythrocyte and leucocytes counts from 0 to 72 h of age

No interaction was found between colostrum and transition feeding protocols for the erythrocyte and leukocytes counts, and no interaction was found for these factors at the time of sampling after birth (Supplementary Table 1). Colostrum and transition feeding protocols did not influence mean erythrocyte count, overall leucocytes count, and for differential mean class counts. Concerning collection time, mean erythrocyte count decreased (*P* < 0.001; Supplementary Fig. S1A), while overall leucocytes count increased until 24 h and declined subsequently (*P* < 0.001; Supplementary Fig. S1B). The monocyte counts increased until 72 h of life (*P* < 0.001; Supplementary Fig. S1C), while the neutrophil count increased in concentration until 24 h with subsequent reduction in the mean count until 72 h of life (*P* < 0.01 Supplementary Fig. S1D).

### Blood parameters from 24 to 72 h of age

The colostrum feeding protocol affected the average total serum protein (TSP) concentration measured from 24 to 72 h (*P* < 0.03; Supplementary Table 2), with calves fed bovine colostrum presenting the higher values.

As for the transition feeding protocol, a trend was observed for calves fed FTM to present higher NEFA concentrations during the first 72 h of life (*P* = 0.07). No interaction between colostrum and transition feeding protocol was observed for all evaluated metabolites (*P* > 0.05). However, TSP, lactate, and NEFA concentrations were influenced by the collection time (*P* < 0.001). TSP concentration decreased from 24 to 72 h (Supplementary Fig. S2A). Glucose was not affected by collection time (Supplementary Fig. S2B). In contrast, lactate concentration decreased from 24 to 48 h and was not different at 72 h (Supplementary Fig. S2C); while NEFA increase from 24 to 48 h and then decreased at 72 h of life (Supplementary Fig. S2D).

### Intake and performance

The colostrum and transition feeding protocols did not affect the calf starter or total dry matter intake (TDMI; Table 2). However, there was an effect of age (*P* < 0.05), and the TDMI increased as calves aged. The intake in the eighth week was also not influenced by treatments. The mean weight, ADG, FE, or body measures (heart girth, hip width, and withers height) were also not influenced by the colostrum or transition feeding protocols, although all of these variables increased over time (*P* < 0.05).

**Table 2:** Preweaning intake, performance, and body measurements of calves receiving different colostrum and transition milk feeding protocols during the first 3d of age.

Item	Treatments				SEM^2^	P-value^3^				
	Bovine colostrum		Enriched colostrum							
	FTM^1^	WM	FTM	WM		C	T	C × T	A	C × T × A
Intake, g DM/day										
Total	940.81	981.88	931.21	970.49	28.448	0.4971	0.1671	0.7231	0.0001	0.3998
Calf starter	202.18	259.12	194.69	228.83	29.915	0.5046	0.1616	0.6465	0.0001	0.4149
Intake at wk 8	567.35	644.74	610.91	633.57	111.492	0.7616	0.3599	0.6000	–	–
Body weight, kg										
At birth	34.46	34.55	34.12	33.87	0.437	0.2464	0.8693	0.6882	–	–
At weaning	65.50	68.53	66.61	67.53	1.480	0.9696	0.1965	0.4656	–	–
ADG^4^, g	556.10	607.50	569.30	603.50	0.027	0.8652	0.1267	0.7455	0.0001	0.5075
FE^5^	0.589	0.598	0.612	0.615	0.026	0.4676	0.8390	0.8958	0.0001	0.5338
Corporal measures, cm										
Heart girth	85.62	85.69	85.69	85.04	0.525	0.6467	0.4733	0.3740	0.0001	0.4158
Hip width	24.43	22.57	22.25	22.46	1.383	0.4755	0.5038	0.5959	0.0001	0.5182
Withers height	83.56	82.58	83.50	83.80	0.781	0.4842	0.3723	0.6342	0.0001	0.8694

^1^Formulated transition milk, Whole milk; ^2^Standard error of the mean; ^3^C = effect of colostrum feeding protocol; T = effect of transition feeding protocol; C × T = effect of interaction between colostrum and transition feeding protocol; A = age effect; C × T × A = effect of interaction between colostrum, transition feeding protocol and age; ^4^Average daily gain; ^5^Feed efficiency.

### Health

The colostrum feeding protocols had no impact on evaluated health variables (Supplementary Table 3). No interaction effects were observed between colostrum and transition milk feeding protocols, and no effects were observed between both protocols and calf age. The fecal score was affected by age of calves (*P* < 0.001; Supplementary Table 3). The highest fecal score values were observed in the second week of life (Supplementary Fig. S3).

### Blood parameters during preweaning

The blood parameters evaluated were all affected by the age of the calves (Table 3), the lowest hematocrit value was observed in the third week (Supplementary Fig. S4), a slight reduction of TSP, a slight increase in glucose, and a decrease in lactate concentrations as calves aged (Supplementary Fig. S5). Only hematocrit was affected by transition feeding protocols (P = 0.007; Table 3). Calves receiving FTM during transition had lower hematocrit compared to calves fed WM. During the preweaning period, there were no effects of colostrum or transition feeding as well as the interaction between these factors for total protein concentration (*P* > 0.05; Table 3). Glucose concentration was affected by an interaction between colostrum and transition feeding protocol (*P* < 0.02), so calves that received bovine colostrum and FTM had higher glucose concentration than those that received enriched colostrum and WM. The feeding of enriched colostrum decreased plasma lactate concentrations (*P* < 0.01), but no effect of the transition feeding protocol was observed.

**Table 3:** Blood parameters of calves receiving different colostrum and transition milk feeding protocols during the preweaning period.

Item	Treatments				SEM^2^	P-value^3^				
	Bovine colostrum		Enriched colostrum							
	FTM^1^	WM	FTM	WM		C^3^	T	C × T	A	C × T × A
Total protein, g/dL	7.26	7.40	7.06	7.24	0.168	0.2843	0.3463	0.9340	0.0001	0.3320
Glucose, mg/dL	128.60^a^	123.67^ab^	125.04^ab^	114.30^b^	3.482	0.0640	0.4094	0.0230	0.0053	0.4554
Lactate, mg/dL	14.20	15.25	13.41	13.63	0.566	0.0185	0.1806	0.6131	0.0001	0.2219
Hematocrit, %	26.07	28.22	25.02	27.12	0.757	0.1565	0.0072	0.9705	0.0001	0.9439

^1^Formulated transition milk, whole milk; ^2^Standard error of the mean; ^3^C = effect of colostrum feeding protocol; T = effect of transition feeding protocol; C × T = effect of interaction between colostrum and transition feeding protocol; A = age effect; C × T × A = effect of interaction between colostrum, transition feeding protocol and age; ^ab^Means within a row with different superscripts are significantly different (P < 0.05).

## Discussion

Both colostrum feeding protocols provided higher immunoglobulin intake than recommended (minimum 300 g IgG) by the current colostrum feeding recommendations for excellent TPI^7^. In our study, both colostrum feeding protocols resulted in immunoglobulin intake greater than 500 g, which resulted in excellent levels of transfer of passive immunity with serum IgG at 48 h of life > 25 g/L and Brix values > 9.4%. Currently, serum IgG concentration higher than 25 g/L is desired, since the higher IgG serum values, the lower the morbidity and mortality rates during the preweaning phase^3,7,30^.

It is well established in the literature that colostrum replacers are efficient in transferring passive immunity when provided in the correct dosage^20,23,31,32^. According to the results in the present study, enriched colostrum was able to provide excellent serum IgG concentrations (> 25 g/L) and serum Brix values (> 9.4%), proving to be a strategy that can help dairy farmers reduce failure of passive transfer in the herd. Transition milk protocols did not influence the transfer of passive immunity variables, as the absorption of immunoglobulins through the intestinal epithelium decreases linearly after birth to complete closure in approximately 24 h^33^. However, prolonged consumption of transition milk brings various benefits, such as reduced morbidity, improved gut development, and performance during preweaning^15,17,18,24,34^.

Regardless of the colostrum feeding protocol, all calves were fed high-quality colostrum (23% to 25% Brix) within the first 3 h of life in the volume corresponding to 12% of birth weight in a single meal. Average AEA values were within the recommended range for calves fed colostrum within a few hours of birth (> 20%)^26^. However, feeding a high mass if IgG in a single colostrum meal might have reduced the AEA^15,23^, decreasing the opportunity to increase IgG blood concentrations even more. Because of that, a second colostrum feeding is recommended^10^. Colostrum feeding through an esophageal tube has been proven to do not negatively affect the AEA when high volume such as 3 L is fed.

Although colostrum feeding was correctly performed, considering IgG mass intake and time after birth, one calf in the bovine colostrum plus FTM group had inadequate TPI, and four other calves—one calf in the bovine colostrum plus FTM group, one in the enriched colostrum plus FTM group, and two calves in the enriched colostrum plus milk group had regular TPI. According to Shivley et al.^35^, 14.2% of calves fed using best colostrum practices (include timing, quality, and quantity) might still have failures in TPI. This failure might be related to calf-dependent factors not currently accounted for, such heat stress during gestation, which might compromise the ability to absorb immunoglobulins^36^ or maintain IgG concentrations after absorption^37^.

The transition feeding protocols were designed to provide 12 L over 3 days, divided into 2 meals of 2 L per day. However, this goal was not met due to the calves' refusal to consume the whole amount provided, mainly calves fed FTM. This result is probably due to the higher solid intake in this transition protocol. In study by Van Soest et al.^24^, providing transition milk (TM) and colostrum replacer added to the milk replacer promoted higher DM intake (862 g/day, and 864 g/day, respectively) compared to the control treatment (milk replacer; 770 g/day). That was also observed in the present study with daily total solids intake of 716 g/day for calves fed FTM and only 480 g/day for calves fed WM. The volume and period of supply of transition milk and its composition are important variables in highlighting the benefits of maternal transition milk or FTM^13,18,37,38^. In other studies, a mixture of 2 L of transition milk and 4L of pasteurized non-commercial milk in the first 21 days of life promoted higher ADG and lower chances of experiencing diarrhea than a control group that received 6 L/day of pasteurized non-commercial milk in the same period^18^. Regarding the volume of milk supplied, Van Soest et al.^24^ showed that supplying approximately 17 L of transition maternal milk or FTM, i.e., 1.9 L/meal, resulted in higher ADG compared to feeding replacer in the first 4 days of life.

Hematological variables showed similar values and trend to those reported for dairy calves of an average of 4 days old^39^. Average TSP was higher for calves fed bovine colostrum when compared to calves that were fed enriched colostrum during the first 72 h, even though there were no differences for IgG concentrations and Brix values at 48 h. This result was not expected since there is a positive correlation between total serum protein and immunoglobulin concentration in the first days of life^40,41^. In addition, there was an increase in total protein concentrations for both colostrum protocols after feeding. Increased circulating NEFA from 24 to 48 h of life might be associated with fat metabolism to meet the metabolic demand for energy and temperature control^42^. However, NEFA concentrations were higher for calves fed FTM, as a result of the higher fat intake, which may improve energy supply for calves and help with thermoregulation^42^.

All colostrum and transition feeding protocols showed higher average fecal scores in the second week of life, but after this period, the values decreased significantly, showing that the calves did not have severe cases of diarrhea, as confirmed by the hematocrit values. The same behavior was observed for antibiotic treatments for respiratory diseases and cattle tick fever. However, providing 280 g/day of colostrum replacer added to whole milk for 3 days was not enough to positively impact health variables, as compared to other studies using transition milk^15,18^. Even though the immunoglobulins in transition milk do not increase IgG absorption due to gut closure around 24 h after birth, these might prevent infections acting at the local gut level against virus and bacteria^3^.

Transition milk comprises bioactive substances, such as lysozyme, lactoferrin, and the lactoperoxidase system, which also contain antimicrobial properties assisting in specific protection against infection^14^. In addition, these compounds have an important action on intestinal development^34^, improving the health and performance of these animals. Thus, animals fed FTM were expected to have superior health variables compared to calves fed only whole milk during the same period. In agreement with the present result, Van Soest et al.^24^ showed that feeding three transition milk protocols for 3 days did not affect health variables. On the other hand, adding 150 g of colostrum replacer to the milk replacer during the first 14 days of life reduced the risk of diarrhea, respiratory illness, umbilical enlargement, and antibiotic use^43^. Kargar et al.^18^, evaluated the effect of replacing pasteurized non-commercial milk with pasteurized transition milk for 3 weeks, concluded that calves receiving 33.33% inclusion of transition milk were less likely to have diarrhea compared to treatments containing 0%, 8.3% or 16.6% transition milk inclusion. These results show that feeding maternal or formulated transition milk for an extended period has greater potential to show benefits than feeding it for a short duration. However, further studies are needed to understand the best feeding strategy, either through larger volumes in a short period after colostrum feeding (3 to 7 days) or through smaller volumes but in a more prolonged manner (14 to 21 days).

Although the calves fed FTM had the lowest average hematocrit values, the values are within the recommended range for cattle, with reference values ranging from 24.3 to 36.6%^44^. Since hematocrit is an indicator of anemia and the degree of dehydration, the present results show that for both transition protocols, the animals were healthy.

The colostrum and transition feeding protocols showed similar starter intake, performance, and body measurements were adequate for all groups. Animals were expected to perform well since all colostrum feeding protocols were designed to provide excellent TPI. Proper colostrum feeding predicts improved performance^45,46^, and every 10 g/L increase in serum IgG concentration after birth was associated with a 2.19 kg increase in body weight at 21 days of age^47^.

Feeding six meals of FTM for 3 days did not improve the performance of calves relative to calves fed whole milk, compared to due to the volume (4 L/day), the number of days (3 days) of feeding FTM, or both. This strategy might not have been sufficient to observe health and performance benefits. Thus, we speculate that the low volume of FTM fed and ingested might have compromised the results of the present study.

In the preweaning period, the different colostrum and transition feeding protocols did not affect TSP concentration. However, a decline in total protein concentration was observed in the second and third week of life, followed by increased concentration with age. This reduction might have occurred due to the decline in colostral immunoglobulins that is common at this age^48^. Subsequently, total protein concentrations begin to increase around the fourth week of age due to endogenous antibody production and the intake of a solid diet, which generates a secondary increase in total protein concentrations^49^.

The glucose concentration evaluated during the preweaning phase showed an interaction effect between colostrum and transition feeding protocol, so feeding bovine colostrum and FTM had higher glucose concentrations than feeding mid-quality enriched colostrum and whole milk. One hypothesis for this interaction would be that colostrum plus FTM promoted higher development of the intestinal epithelium favoring increased glucose absorption, even though no differences were observed for performance. Maternal colostrum feeding promotes increased villus size and reduced crypt depth^34^, improving glucose absorption in calves in the first days of life^50,51^. Perhaps these effects can also be observed in the long term, as in the present work.

In the present study, calves fed bovine colostrum had higher lactate concentrations than calves fed enriched colostrum. The lactate concentration in the bloodstream might originate through fermentation and metabolism of propionate in the ruminal wall^52^, and is stimulated by the intake of a solid diet, but the consumption of concentrate was similar between colostrum feeding protocols. Furthermore, lactate concentrations decreased with age for both colostrum feeding protocols.

In conclusion, enriching mid-quality colostrum by adding colostrum replacer is a suitable alternative in situations with a shortage of maternal colostrum in adequate quality for the newborn, resulting in similar passive transfer as high-quality bovine colostrum. However, providing low-volume (4 L/day) of formulated transition milk (70 g colostrum replacer/L) for a short time (3 days) does not provide additional health and performance benefits to calves in the short term. More data regarding to transition milk nutrient and bioactive compounds composition are needed to better define meal size and feeding period to improve health and performance of calves.

### Supplementary Information

Supplementary Information.

## Data Availability

The datasets used and/or analyzed during the current study are available from the corresponding author on reasonable request.
