# Supplementary material for: Enrichment of medium-quality colostrum by adding colostrum replacer, combined or not with transition milk in the feeding of dairy calves
Source: Sci Rep. 2024 Mar 6;14:5533. doi: 10.1038/s41598-024-55757-4 (PMC10917790; doi:10.1038/s41598-024-55757-4)
Supplement: Supplementary file 1 — Supplementary Information. [file 41598_2024_55757_MOESM1_ESM.docx]

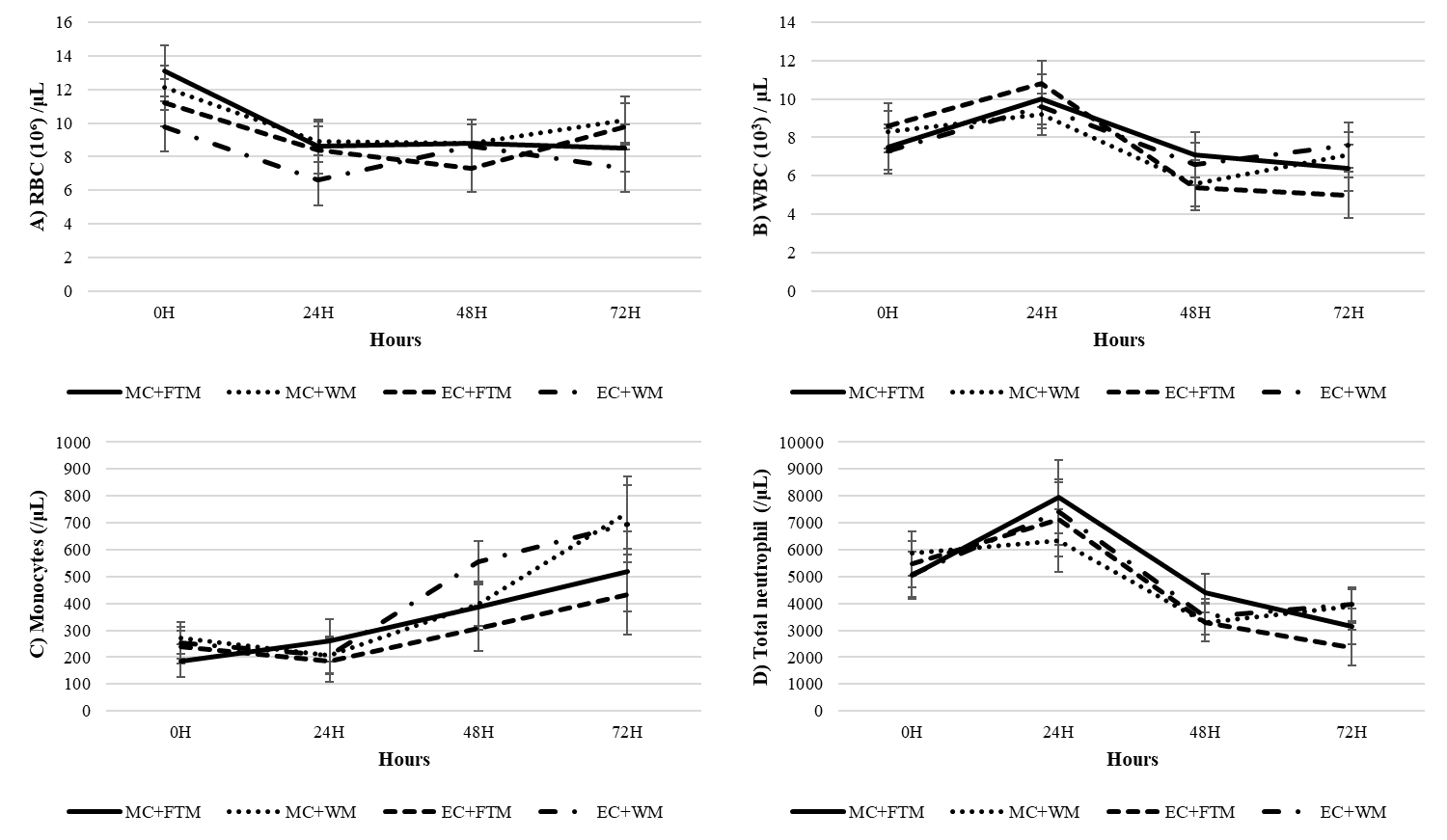


**Supplementary Figure S1**. Effect of hours of life on (A) red blood cell (RBC), (B) overall white blood cell (WBC) count, (C) monocyte, (D) neutrophil counts of calves receiving different colostrum and transition milk feeding protocols in the first 72 hours of life. MC+FTM, Bovine maternal colostrum + formulated transition milk; MC+WM, Bovine maternal colostrum + whole milk; EC+FTM, Enriched colostrum + formulated transition milk; EC+WM, Enriched colostrum + whole milk


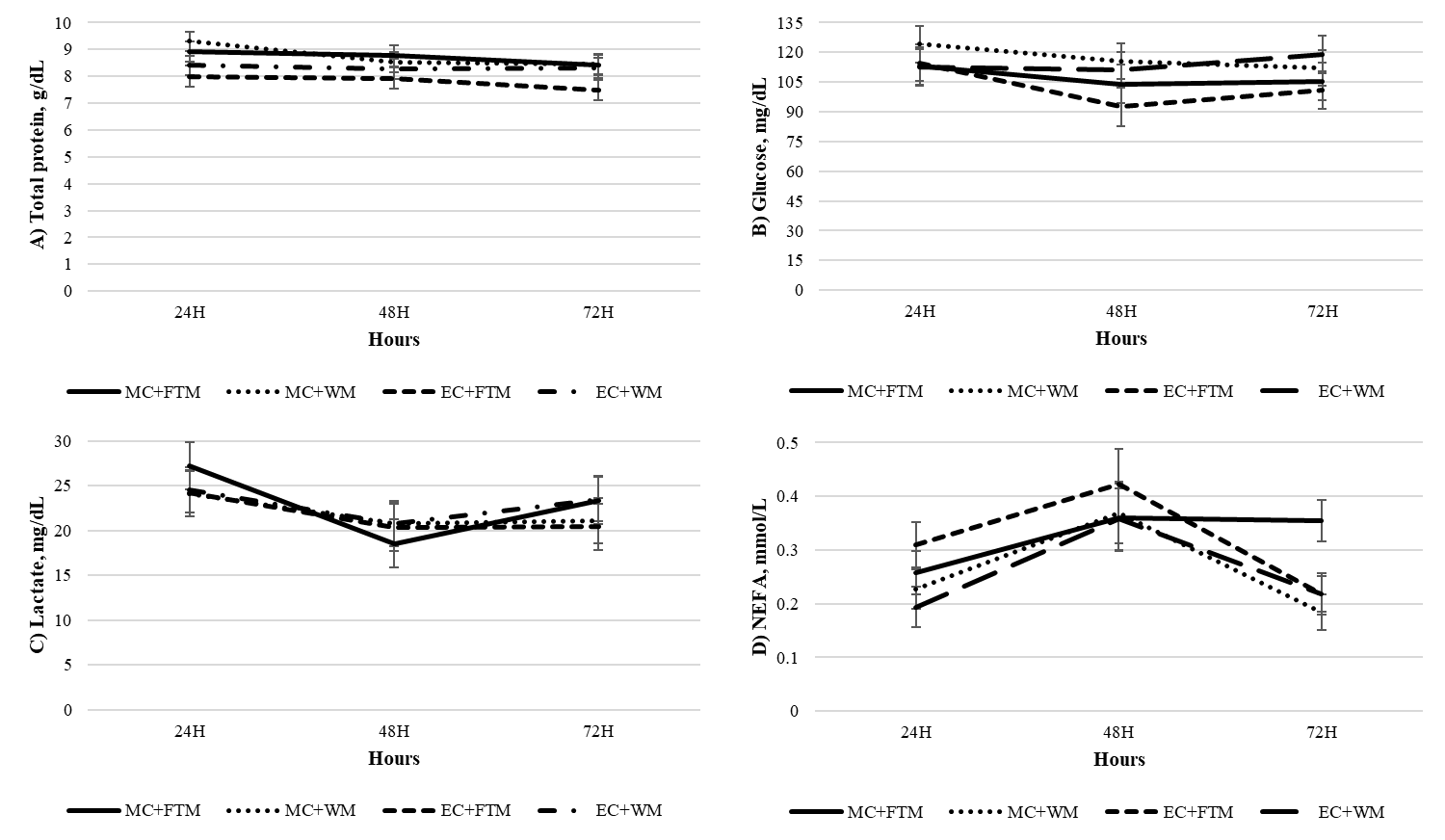


**Supplementary Figure S2**. Effect of age on concentrations of (A) Total protein; (B) Glucose; (C) Lactate; (D) NEFA of calves receiving different colostrum and transition milk feeding protocols in the first 72 hours of life. MC+FTM, Bovine maternal colostrum + formulated transition milk; MC+WM, Bovine maternal colostrum + whole milk; EC+FTM, Enriched colostrum + formulated transition milk; EC+WM, Enriched colostrum + whole milk


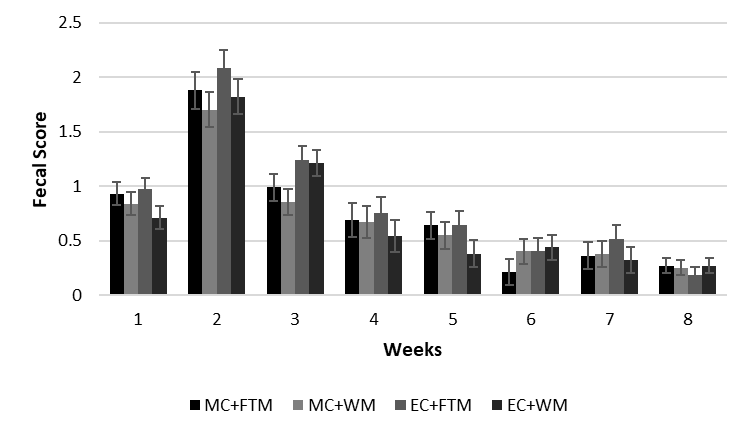


**Supplementary Figure S3**. Fecal score of calves receiving different colostrum and transition milk feeding protocols during the preweaning period. MC+FTM, Bovine maternal colostrum + formulated transition milk; MC+WM, Bovine maternal colostrum + whole milk; EC+FTM, Enriched colostrum + formulated transition milk; EC+WM, Enriched colostrum + whole milk


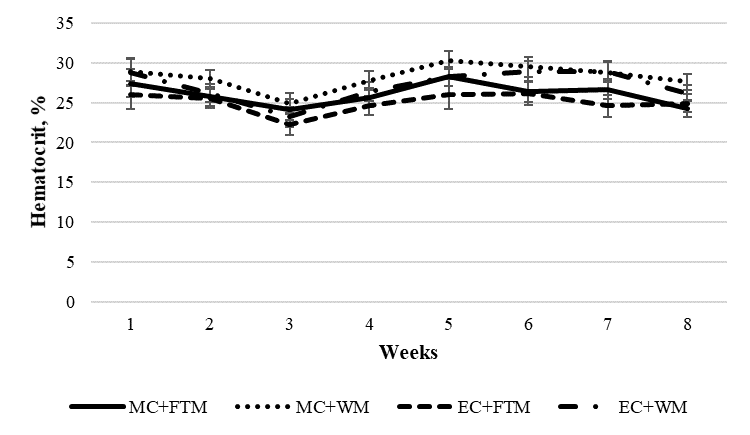


**Supplementary Figure S4**. Percent hematocrit of calves receiving different colostrum and transition milk feeding protocols during the preweaning period. MC+FTM, Bovine maternal colostrum + formulated transition milk; MC+WM, Bovine maternal colostrum + whole milk; EC+FTM, Enriched colostrum + formulated transition milk; EC+WM, Enriched colostrum + whole milk


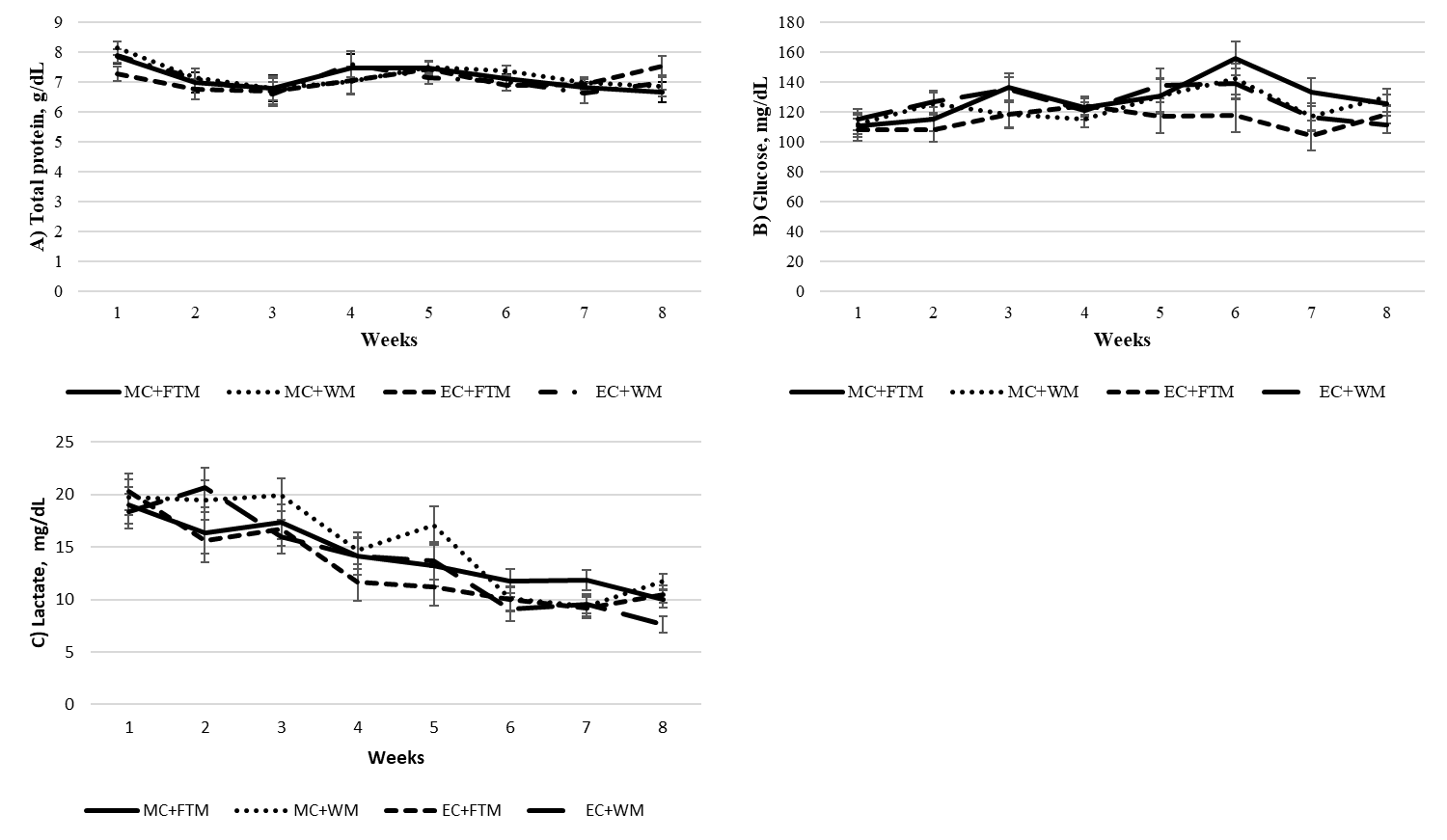


**Supplementary Figure S5.** Blood parameters of calves receiving different colostrum and transition milk feeding protocols during the preweaning period. (A) Total protein; (B) Glucose; (C) Lactate. MC+FTM, Bovine maternal colostrum + formulated transition milk; MC+WM, Bovine maternal colostrum + whole milk; EC+FTM, Enriched colostrum + formulated transition milk; EC+WM, Enriched colostrum + whole milk

**Supplementary Table 1**. Mean erythrocyte and total and differential leukocyte counts in the first 72 hours of life of calves receiving different colostrum and transition milk feeding protocols.

| Item | Treatments | | | |  | P-value^3^ | | | | |
| --- | --- | --- | --- | --- | --- | --- | --- | --- | --- | --- |
|  | Maternal colostrum | | Enriched colostrum | | SEM^2^ |  |  |  |  |  |
|  | FTM^1^ | WM | FTM | WM |  | C | T | C x T | H | C x T x H |
| Erythrocytes, x10⁶/μL | 9.7 | 10.0 | 9.2 | 8.4 | 8.39 | 0.179 | 0.725 | 0.480 | 0.002 | 0.767 |
| Leukocytes, 10^3^/μL | 7.8 | 7.4 | 7.1 | 7.7 | 6.92 | 0.750 | 0.852 | 0.434 | < 0.001 | 0.477 |
| Eosinophils, /μL | 15.7 | 38.8 | 39.4 | 33.0 | 13.59 | 0.487 | 0.516 | 0.244 | 0.315 | 0.407 |
| Basophil, /μL | 5.9 | 13.2 | 9.3 | 12.9 | 6.18 | 0.787 | 0.365 | 0.754 | 0.126 | 0.181 |
| Lymphocytes, /μL | 2271.1 | 2139.1 | 2332.9 | 2259.9 | 235.43 | 0.655 | 0.619 | 0.882 | 0.150 | 0.212 |
| Monocytes, /μL | 338.2 | 403.8 | 291.2 | 427.3 | 46.00 | 0.822 | 0.105 | 0.493 | < 0.001 | 0.615 |
| Neutrophils, /μL | 5136.0 | 4852.1 | 4564.1 | 4990.0 | 517.97 | 0.637 | 0.875 | 0.425 | < 0.001 | 0.500 |

^1^Formulated transition milk, Whole milk; ^2^Standard error of the mean; ^3^C = effect of colostrum feeding protocol; T = effect of transition feeding protocol; C x T = effect of interaction between colostrum and transition feeding protocol; C x T x H = effect of interaction between colostrum, transition feeding protocol and hour;

**Supplementary Table 2.** Blood parameters in the first 72 hours of life of calves receiving different colostrum and transition milk feeding protocols

| Item | Treatments | | | |  | P-value^3^ | | | | |
| --- | --- | --- | --- | --- | --- | --- | --- | --- | --- | --- |
|  | Maternal colostrum | | Enriched colostrum | | SEM^2^ |  |  |  |  |  |
|  | FTM^1^ | WM | FTM | WM |  | C | T | C x T | H | C x T x H |
| Total protein, g/dL | 8.70 | 8.75 | 7.80 | 8.32 | 0.297 | 0.0295 | 0.3455 | 0.4322 | 0.0483 | 0.7630 |
| Glucose, mg/dL | 107.3 | 117.2 | 102.8 | 114.1 | 6.59 | 0.5660 | 0.1166 | 0.9079 | 0.1779 | 0.8153 |
| Lactate, mg/dL | 23.05 | 22.01 | 21.64 | 22.92 | 1.635 | 0.8757 | 0.9432 | 0.4981 | 0.0169 | 0.8889 |
| NEFA^4^, mmol/L | 0.324 | 0.260 | 0.316 | 0.256 | 0.0332 | 0.8669 | 0.0706 | 0.9559 | <0.001 | 0.1513 |

^1^Formulated transition milk, Whole milk; ^2^Standard error of the mean; ^3^C = effect of colostrum feeding protocol; T = effect of transition feeding protocol; C x T = effect of interaction between colostrum and transition feeding protocol; H = hour effect; C x T x H = effect of interaction between colostrum, transition feeding protocol and hour; ^4^Non-esterified fatty acids

**Supplementary Table 3**. Fecal score and health of preweaning calves receiving different colostrum and transition milk feeding protocols in the first 3d of life.

| Item | Treatments | | | |  | P-value^3^ | | | | |
| --- | --- | --- | --- | --- | --- | --- | --- | --- | --- | --- |
|  | Maternal colostrum | | Enriched colostrum | | SEM^2^ |  |  |  |  |  |
|  | FTM^1^ | WM | FTM | WM |  | C | T | C x T | A | C x T x A |
| Fecal score | 0.75 | 0.71 | 0.83 | 0.71 | 0.057 | 0.4298 | 0.1784 | 0.4713 | 0.0001 | 0.4435 |
| Days with pyrexia | 4.03 | 3.31 | 4.73 | 3.21 | 0.760 | 0.6949 | 0.1524 | 0.5881 | - | - |
| Days with diarrhea | 9.13 | 7.23 | 10.09 | 8.51 | 1.175 | 0.3427 | 0.1523 | 0.8904 | - | - |
| Number of treatments | | |  |  |  |  |  |  |  |  |
| ATB^4^ | 1.26 | 0.73 | 1.33 | 0.99 | 0.264 | 0.5269 | 0.1092 | 0.7057 | - | - |
| Diarrhea | 0.06 | 0.05 | 0.26 | 0.06 | 0.137 | 0.2867 | 0.2953 | 0.3163 | - | - |
| RD^5^ | 0.27 | 0.22 | 0.36 | 0.15 | 0.110 | 0.9559 | 0.2423 | 0.4580 | - | - |
| CTF^6^ | 0.72 | 0.38 | 0.70 | 0.87 | 0.191 | 0.2178 | 0.6614 | 0.1789 | - | - |

1Formulated transition milk, Whole milk; ^2^Standard error of the mean; ^3^C = effect of colostrum feeding protocol; T = effect of transition feeding protocol; C x T = effect of interaction between colostrum and transition feeding protocol; A = age effect; C x T x A = effect of interaction between colostrum, transition feeding protocol and age; ^4^Antibiotic; ^5^Respiratory disease; ^6^Cattle tick fever
